# Supplementary material for: Mutation of the Drosophila melanogaster serotonin transporter dSERT impacts sleep, courtship, and feeding behaviors
Source: PLoS Genet. 2022 Nov 21;18(11):e1010289. doi: 10.1371/journal.pgen.1010289 (PMC9721485; doi:10.1371/journal.pgen.1010289)
Supplement: S1 Fig — Hourly sleep traces (A) and quantification of total sleep (B) in w1118 controls (grey), transheterozygous dSERT10/dSERT16 mutants (yellow), homozygous dSERT10 mutants (orange), and homozygous dSERT16 mutants (red). Hourly sleep traces (C) and quantification of total sleep (D) in w1118 controls (grey), dSERT16 heterozygotes (purple), and dSERT16 homozygous mutants (red). Hourly sleep traces (E) and quantification of total sleep (F) in w1118 controls (grey), dSERT4 revertant (light blue), d04388 parental, P element line (teal), and dSERT16 mutants (red). (G-H) Quantification of P(Wake) (G) and P(Doze) (H) during the light period (LP, Zeitgeber hours 1–12) and dark period (DP, Zeitgeber hours 13–24) in w1118 controls (grey), dSERT10 mutants (orange), and dSERT16 mutants (red). For all panels, sleep traces show mean ± SEM and histograms show both individual datapoints and group means ± SEM. One way ANOVA with Tukey post-hoc test (p≤0.0332*, p≤0.0021**, p≤0.0002***, p≤0.0001****). LP and DP were analyzed separately in G, H. (PDF) [file pgen.1010289.s001.pdf]

Supplemental Figure 1

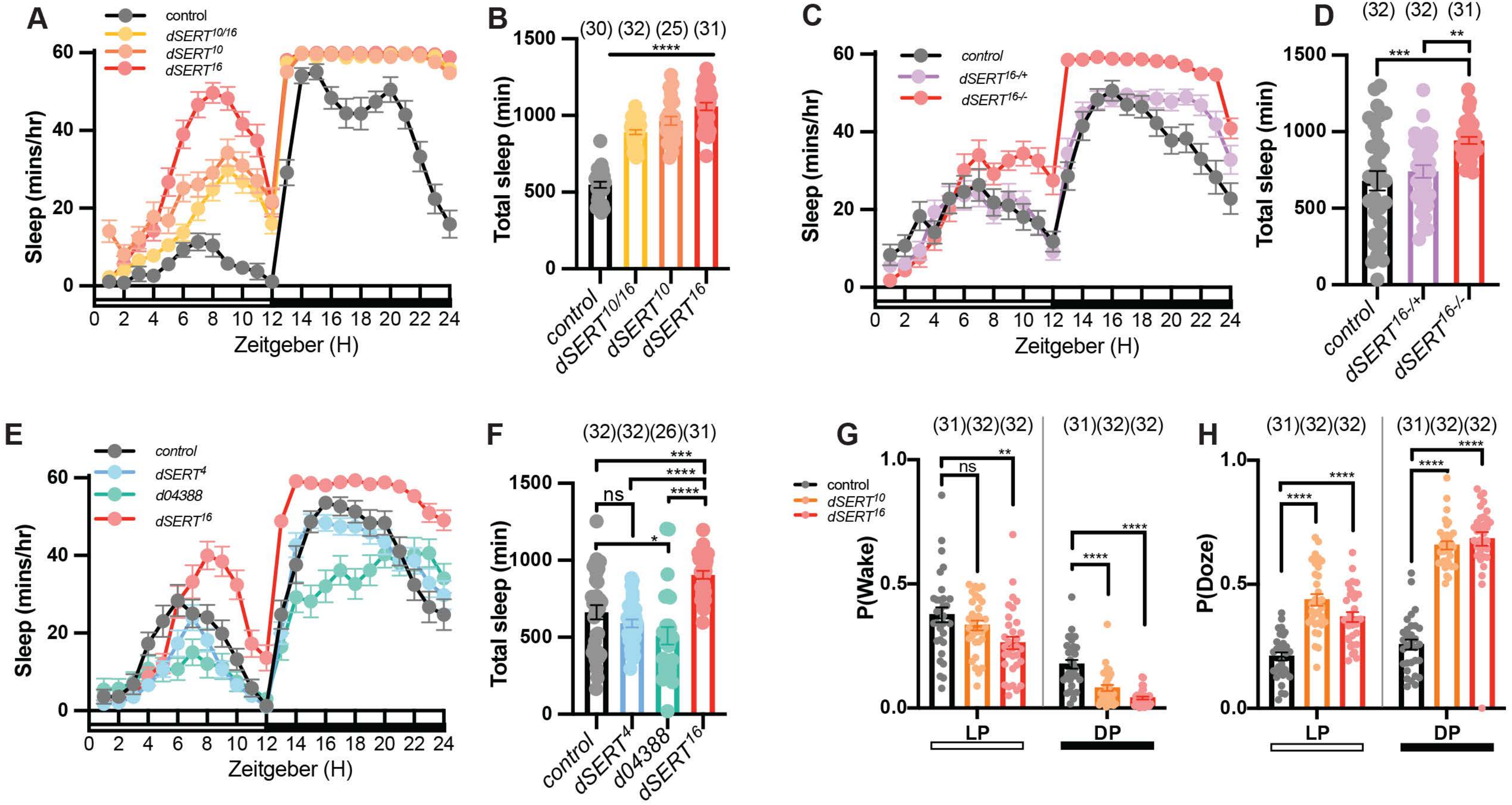

Supplemental Figure 1. Genetic controls, P(doze) and P(wake). Hourly sleep traces (A) and quantification of total sleep (B) in w^{1118} controls (grey), transheterozygous $dSERT^{10}/dSERT^{16}$ mutants (yellow), homozygous $dSERT^{10}$ mutants (orange), and homozygous $dSERT^{16}$ mutants (red). Hourly sleep traces (C) and quantification of total sleep (D) in w^{1118} controls (grey), $dSERT^{16}$ heterozygotes (purple), and $dSERT^{16}$ homozygous mutants (red). Hourly sleep traces (E) and quantification of total sleep (F) in w^{1118} controls (grey), $dSERT^{4}$ revertant (light blue), *d04388* parental, *P* element line (teal), and $dSERT^{16}$ mutants (red). (G-H) Quantification of P(Wake) (G) and P(Doze) (H) during the light period (LP, Zeitgeber hours 1-12) and dark period (DP, Zeitgeber hours 13-24) in w^{1118} controls (grey), $dSERT^{10}$ mutants (orange), and $dSERT^{16}$ mutants (red). For all panels, sleep traces show mean ± SEM and histograms show both individual datapoints and group means ± SEM. One way ANOVA with Tukey post-hoc test ($p \leq 0.0332$*, $p \leq 0.0021$**, $p \leq 0.0002$***, $p \leq 0.0001$****). LP and DP were analyzed separately in G, H.
