# Supplementary material for: Mutation of the Drosophila melanogaster serotonin transporter dSERT impacts sleep, courtship, and feeding behaviors
Source: PLoS Genet. 2022 Nov 21;18(11):e1010289. doi: 10.1371/journal.pgen.1010289 (PMC9721485; doi:10.1371/journal.pgen.1010289)
Supplement: S2 Fig — (A) dSERT16 mutants (purple) show no change in grooming behavior compared to w1118 controls (grey). The average number of grooming events per minute for three separate 2 min periods with three experimental replicates is shown. In each experimental replicate n = 5 flies for each genotype. Mean± SEM, unpaired Student’s t-test. (B) Male and female dSERT16 mutants behave indistinguishably from control flies in negative geotaxis assays. Mean ± SEM, one-way ANOVA. (PDF) [file pgen.1010289.s002.pdf]

Supplementary Figure 2

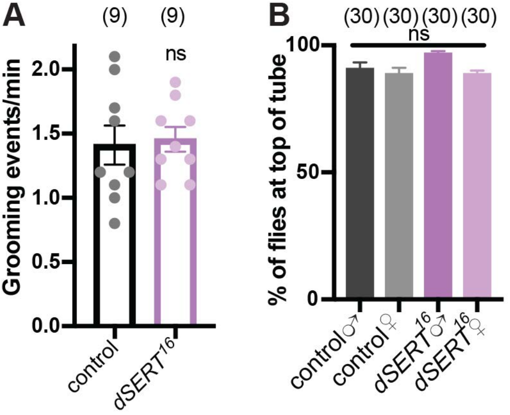

Supplemental Figure 2. ***dSERT^{16}* mutant sleep phenotype is not an artifact of additional amine-linked behaviors.** (A) *dSERT^{16}* mutants (purple) show no change in grooming behavior compared to *w^{1118}* controls (grey). The average number of grooming events per minute for three separate 2 min periods with three experimental replicates is shown. In each experimental replicate n= 5 flies for each genotype. Mean± SEM, unpaired Student's t-test. (B) Male and female *dSERT^{16}* mutants behave indistinguishably from control flies in negative geotaxis assays. Mean ± SEM, one-way ANOVA.
