# Supplementary material for: Mutation of the Drosophila melanogaster serotonin transporter dSERT impacts sleep, courtship, and feeding behaviors
Source: PLoS Genet. 2022 Nov 21;18(11):e1010289. doi: 10.1371/journal.pgen.1010289 (PMC9721485; doi:10.1371/journal.pgen.1010289)
Supplement: S3 Fig — (A) Quantification of total sleep in w1118 and dSERT16 males (blue), virgin females (pink), or mated females (red). Analysis of daytime sleep (B) and daytime bout frequency (C) and latency (D). Quantification of nighttime sleep (E) and nighttime bout number (F). Graphs show individual datapoints and group means ± SEM. Two-way ANOVA, with Tukey post-hoc test (p≤0.0332*, p≤0.0002***, p≤0.0001****). (PDF) [file pgen.1010289.s003.pdf]

Supplemental Figure 3

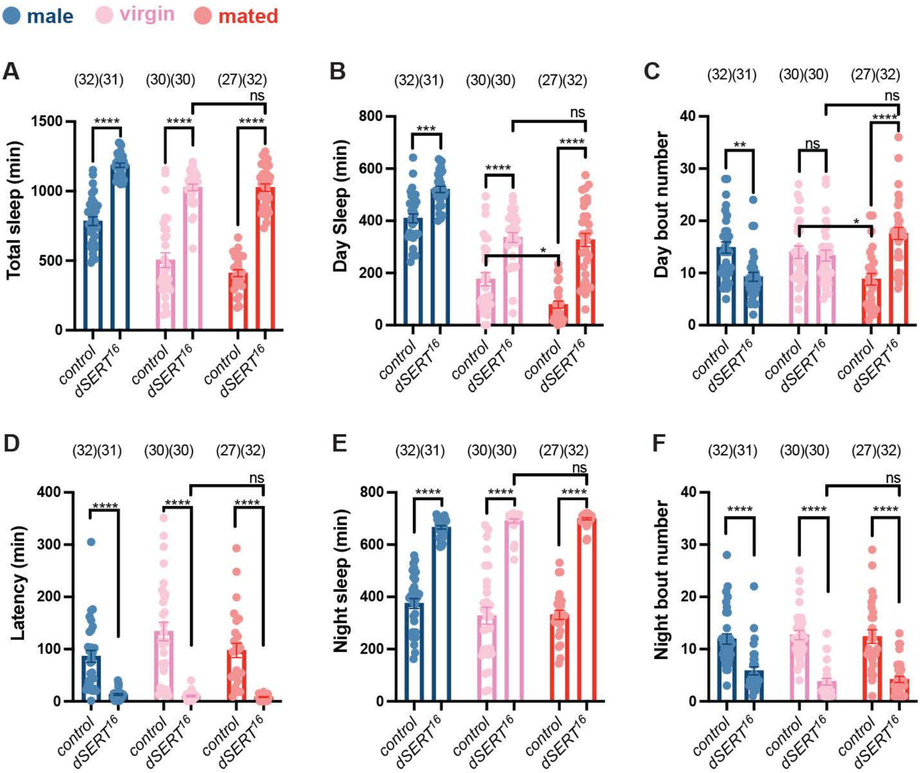

Supplemental Figure 3. Sleep in *dSERT* mutants of different genders and mating status. (A) Quantification of total sleep in w^{1118} and $dSERT^{16}$ males (blue), virgin females (pink), or mated females (red). Analysis of daytime sleep (B) and daytime bout frequency (C) and latency (D). Quantification of nighttime sleep (E) and nighttime bout number (F). Graphs show individual datapoints and group means ± SEM. Two-way ANOVA, with Tukey post-hoc test ($p \leq 0.0332$*, $p \leq 0.0002$***, $p \leq 0.0001$****).
