# Supplementary material for: Mutation of the Drosophila melanogaster serotonin transporter dSERT impacts sleep, courtship, and feeding behaviors
Source: PLoS Genet. 2022 Nov 21;18(11):e1010289. doi: 10.1371/journal.pgen.1010289 (PMC9721485; doi:10.1371/journal.pgen.1010289)
Supplement: S4 Fig — Proportion of w1118 (black) and dSERT16 (red) flies awakened after 5 seconds of either 0.5 g or 1 g vibrational stimulation during the daytime (A) or nighttime (B) (n = 24 trials per groups, n = 30–32 flies per group). Mean± SEM, Two-way ANOVA with Tukey post-hoc test (≤0.0021**). (PDF) [file pgen.1010289.s004.pdf]

Supplemental Figure 4

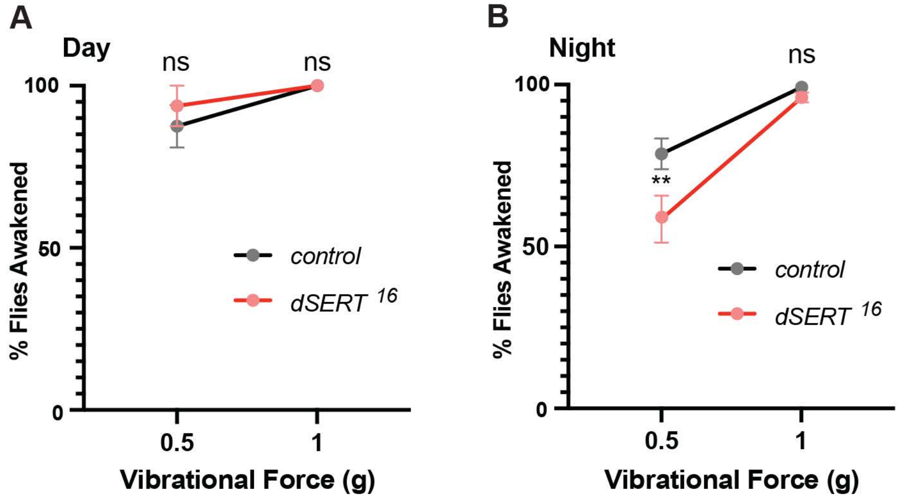

Supplemental Figure 4. Arousal threshold of *dSERT*^{16} flies. Proportion of *w*^{1118} (black) and *dSERT*^{16} (red) flies awakened after 5 seconds of either 0.5 g or 1 g vibrational stimulation during the daytime (A) or nighttime (B) (n=24 trials per groups, n=30-32 flies per group). Mean± SEM, Two-way ANOVA with Tukey post-hoc test (≤0.0021**).
