# Supplementary material for: Mutation of the Drosophila melanogaster serotonin transporter dSERT impacts sleep, courtship, and feeding behaviors
Source: PLoS Genet. 2022 Nov 21;18(11):e1010289. doi: 10.1371/journal.pgen.1010289 (PMC9721485; doi:10.1371/journal.pgen.1010289)
Supplement: S5 Fig — (A-B) Representative pictures show expression of UAS-MCD8::GFP (green) driven by TRH-Gal4 (A) or “TPH”-Gal4 (B) and labeled with an antibody to DLG (magenta) in mushroom bodies. Mushroom body lobes are labeled with white text. (PDF) [file pgen.1010289.s005.pdf]

Supplemental Figure 5

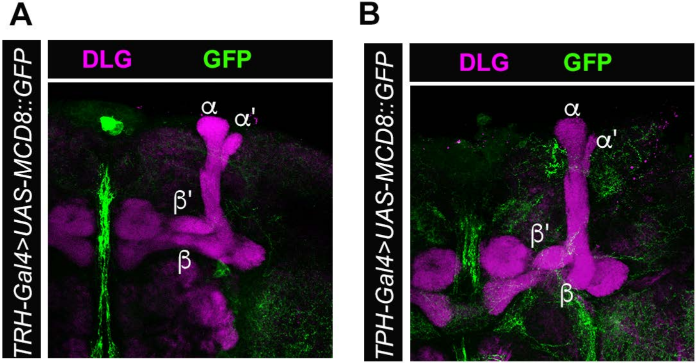

Supplemental Figure 5. (A-B) Representative pictures show expression of UAS-MCD8::GFP (green) driven by *TRH-Gal4* (A) or "*TPH"-Gal4* (B) and labeled with an antibody to DLG (magenta) in mushroom bodies. Mushroom body lobes are labeled with white text.
