# Supplementary material for: Mutation of the Drosophila melanogaster serotonin transporter dSERT impacts sleep, courtship, and feeding behaviors
Source: PLoS Genet. 2022 Nov 21;18(11):e1010289. doi: 10.1371/journal.pgen.1010289 (PMC9721485; doi:10.1371/journal.pgen.1010289)
Supplement: Attachment — Submitted filename: Response to Reviewers 10 14 22 M acpt chngs submitted.docx [file pgen.1010289.s007.docx]

We thank both the Editor and the Reviewers for excellent constructive criticism and for the opportunity to submit a revised manuscript. As described below, we have performed a number of new experiments to address the Reviewers’ concerns, added a new main figure, revised 8 of the 9 previous figures and added 2 supplemental figures. We have also revised the text, and the changes are indicated in Track Changes. As suggested, we have also modified the title to “Mutation of the *Drosophila melanogaster* serotonin transporter dSERT impacts sleep, courtship, and feeding behaviors”. Our detailed response to each of the Reviewers’ comments is listed below.

***Reviewer 1:***

Major Concerns:

*1. As we know that, flies have sexual dimorphism in sleep behavior. The authors monitored and analysis the sleep properties of female flies with wild-type or SerT mutant genotype. Did the authors use virgin females? How about the males? The authors should also describe the gender used in RNA and protein experiments.*

For the majority of the sleep experiments, and unless noted otherwise in co-housing experiments, we used mated, female flies in our original manuscript. We now include this important parameter in the Materials and Methods section of the revised manuscript. We have also updated the Methods section to more clearly state the gender used for qRT-PCR and Westerns.

We agree with the Reviewer that exploring the differential effects of *dSERT* on sleep behavior in males versus females and in virgin versus mated females is important. Therefore, we performed additional experiments to compare the sleep phenotype of males, virgin females, and mated females, testing both controls and *dSERT^1^*^6^ in parallel. Our findings are included in Supplemental Figure 3 of the revised manuscript. The data suggest that similar to mated females, male and virgin females of *dSERT* mutants also exhibit the same significant increase in total sleep, daytime sleep, and nighttime sleep compared to controls. We also see reduced latency to sleep and a consistent consolidation of sleep bouts at night in all three groups. By contrast, we observe a difference in daytime architecture between the three groups, with only mated females showing an *increase* in daytime bout number (Supplemental Figure 3C). By contrast, *dSERT^1^*^6^ males show a decrease in day bout number compared to controls, and we do not detect a difference between unmated *dSERT^1^*^6^ females and controls. The sex peptide mediated post mating response includes a decrease daytime sleep in mated females^1^. The increase in day bout number in mated *dSERT^1^*^6^ females may reflect an increase in activity similar to the decrease in sleep seen with the post-mating response. If so, it is possible that the circuits regulated by sex peptide and serotonin could interact. We will consider exploring this possibility in future experiments.

In the text we now state:

“Interestingly, in contrast to nighttime sleep architecture, the effects of *dSERT^16^* on *daytime* architecture differed between the three groups; mated *dSERT* females showed an increase in daytime bout number (frequency) compared to controls that was not observed in either males or virgin females (Sup Figure 3C).”

*2. In co-housing experiments, the authors put one male and one female flies in a tube to monitor their sleep. However, only one male or female was monitored as control group. To exclude the possibility that two flies disrupt each other instead of copulation, or the monitoring deviation, the author should put two male or female flies in a tube as control.*

We agree, and to correct this oversight we have added additional controls. In the revised manuscript we include in Figure 5A (open or shaded dark red bar) data using two females co-housed together as a control (indicated as “♀+♀” on the x axis).

*3. The author presents multiple behaviors in SerT mutant flies. They showed reduced food intake and didn’t have the effect of starvation induced sleep loss. It may be due to the possibility that sleep increase reduce the foraging time. They also showed reduced courtship and copulation behaviors. It is likely that the waking threshold was elevated in SerT mutant. I would suggest that it will be better to make waking threshold tests, which will be easy to explain those changing in distinct behaviors.*

We thank the Reviewer for this suggestion. In response, we performed additional experiments to assay the waking threshold of controls versus *dSERT^16^* mutants. We used an automated method to periodically give a mechanical stimulus (vibration) to the flies and compared the effects of two stimuli of different strengths: 0.5 and 1.0 x the gravitational constant (g). The data from these experiments are shown in Supplemental Figure 4 of the revised manuscript. They show that, at night, the *dSERT* mutant has a modest but statistically significantly elevation in waking threshold compared to controls. This is seen as a lower percentage of mutant lies awakened with the less intense (0.5 g) stimulus. Consistent with the Reviewer’s suggestion, the elevation in waking threshold could contribute to the increase in *dSERT* mutant’s sleep at night during starvation or co-housing. In addition to Supplemental Figure 4, we have added text describing this effect to the revised manuscript:

“We did not detect any differences in arousability between control and *dSER*T mutants when they were mechanically stimulated during the day using either stimulus (Sup Figure 4A) or during the night using the stronger stimulus (1.0 g). By contrast, *dSERT* mutants exhibited a significant decrease in arousability compared to controls when exposed to a less intense vibrational stimulus (0.5 g) (Sup Figure 4B). These data suggest, that at night, *dSERT* mutants have a somewhat elevated arousal threshold, and an increased intensity of sleep. This decrease in arousability may contribute to the lack of wakefulness seen during starvation and co-housing conditions.”

*4. The author used two gal4 lines, TRH-gal4 and TPH-gal4, to rescue the sleep phenotype caused by SerT mutant. Interestingly, the two gal4 lines rescued different aspect of sleep properties of SerT mutant. I am curious about whether the combination of the two gal4s could rescue the all the defects caused by SerT mutant. If it’s not easy to manipulate, how about a broader gal4, such as a pan-neural gal4??*

We thank the Reviewer for this excellent suggestion. In response, we performed an additional rescue experiment in which both gal4s (*TPH-Gal4* and *TRH-Gal4*) were used to express *UAS-dSERT.* We have included the data in our revised manuscript as Figure 7G-I. As predicted, the combination of both gal4s is sufficient to rescue sleep in both daytime and nighttime periods.

We have added the following text to the Results:

“We next tested whether combining the serotonergic drivers could fully restore sleep levels across the 24-hour period. Our results show that genetic rescue using both *TPH-Gal4* and *TRH-Gal4* to express *UAS-dSERT* in the *dSERT* mutant is sufficient to restore sleep levels to that of the controls in both the daytime and nighttime (Figure 7G-I).”

*Did the activation or silencing of neurons using TRH-gal4 or TPH-gal4 caused similar sleep phenotype of SerT mutant, respectively?*

This in an interesting idea since activation of serotonergic neurons would be predicted to phenocopy *dSERT^16^* and disrupt sleep. Another group has indeed used *TPH-Gal4* or *TRH-Gal4* to express *UAS-TRPA1* to briefly activate serotonergic neurons and reported a transient period characterized as “behavioral quiescence” ^2^. Since the effects of extended activation were not reported, it was not clear how this might affect sleep. However, more recently, the Griffith lab demonstrated that *TRH-Gal4>UAS-TRPA1* activation of serotonergic neurons induced an increase in bout frequency and sleep fragmentation^3^, a phenotype consistent with disruption of sleep. Since these experiments are similar to those suggested by the Reviewer and have already been published, we did not include additional data on manipulating the activity of serotonergic neurons in our revised manuscript.

*5. The authors mentioned frequently that dSERT mutant akin to the clinical side effects of SSRIs, they should design experiments, such as using drugs to alter circular 5HT, or the authors should minor the descriptions.*

To address this important concern, we performed additional experiments in which we fed wildtype flies the SSRI citalopram and assayed sleep. The data are shown as Figure 3 of our revised manuscript. We find that WT flies fed citalopram exhibit a dose-dependent increase in sleep during both the daytime and nighttime periods, thereby phenocopying the *dSERT* mutant as predicted. In addition to Figure 3, we have added the following text:

“To further confirm the increased sleep is a result of disrupted dSERT function, we tested whether feeding adult flies an SSRI to block dSERT activity would impact sleep. *w^1118^* flies fed the SSRI citalopram showed a dose dependent increase in total sleep (Figure 3A). Consistent with our findings with the *dSERT* mutants, both daytime sleep (Figure 3C) and nighttime sleep (Figure 3D) were significantly increased in wildtype flies fed citalopram. We do not detect any further increase in sleep in *dSERT^16^* mutants fed citalopram (Figure 3B) supporting the idea that citalopram’s effect on sleep is mediated by inhibition of dSERT rather than a spurious, off-target effect. In addition, these findings indicate that an increase in adult sleep can be caused by disrupting dSERT only in the adult and not during development. However, further experiments will be needed to determine whether additional, and possibly more subtle developmental defects may occur in the mutants.”

*Particularly, the authors demonstrated in Fig.6, overexpression of dSERT in TRH-GAL4, flies showed no difference compared with control.*

As noted by the Reviewer, we did not observe any effect of *dSERT* overexpression. An increase in SERT expression could in principle have an effect opposite to that caused by blocking SERT activity, either with a mutation or an SSRI. Since we did not observe any effect, we speculate that serotonin reuptake may be functionally maximal with WT levels of dSERT, thus negating the possibility that an increase in expression could detectably increase activity. We note however, that the behavioral assays used in our experiments may not be sensitive enough to detect more subtle neurochemical changes. Additional experiments using cyclic voltammetry or a molecular sensor would be necessary to explore this possibility but are beyond the scope of the current report.

Minor concerns:
*1. Please carefully recheck the font in the text (such as line 228), misplace in the figures (such as Figure 3) and mistakes in the methods (such as H20, NaCL, “I will add food recipe”).*

We regret these unfortunate errors and have edited the text to correct them.

*2. The behavior of flies, especially sleep, is sensitive to the genetic background. The author backcrossed the P-element line to w1118. However, the cross to [Δ2-3] line and the imprecise excision may also change the genomic background. The author should backcross the flies after the generation of SerT10 and SerT16 lines.*

We agree that genetic background can have profound effects on behavior and appreciate the Reviewer’s point. However, redoing all of our experiments would make it difficult to revise our manuscript in a timely manner. Therefore, we have addressed this important concern with additional data. We compared sleep behavior between *w^1118^*, used as the control in most of our experiments, with a precise excision of the P element (*dSERT^4^*) (see Supplemental Figure 1E-F of the revised manuscript). Since *dSERT^4^* was obtained in the screen that yielded the imprecise excisions, it is in the exact same genetic background as *dSERT^10^ and dSERT^16^* . We also compared the sleep behavior of *dSERT^4^* to that of *dSERT^16^ _._* We did not detect any difference between sleep behavior of *w^1118^* and *dSERT^4^* but see a clear increase in *dSERT^16^* compared to *dSERT^4^* (Supplemental Figure 1E-F). We conclude that the genetic background is unlikely to be responsible for the sleep phenotype of the d*SERT* imprecise excision mutants. By contrast, we observe a slight decrease in sleep in the parent P element line (*d04388*) compared to controls (Supplemental Figure 1E-F). As suggested by the Reviewer, the difference between the overall genetic architecture of the parent line versus the excision lines could contribute to sleep behavior. It is also possible that positional effects of the P element could affect *dSERT* expression, e.g., by changing the developmental timing, level or pattern of *dSERT* expression. We have not pursued additional studies of the parent line since it would be difficult to distinguish between these possibilities and it is unclear how the results would relate to the clear loss of *dSERT* expression we see in the imprecise excision lines.

In addition to the data in Supplemental Figure 1, we have added the following text to the revised manuscript:

“To determine how genetic background may contribute to the behavior we observed, sleep in *dSERT^16^* was also compared to the revertant *dSERT^4^* control, which was obtained in the same screen as the imprecise excisions and shares the same genetic background. We also assayed sleep in the parental P-element line *P{XP}d04388* (Sup Figure 1E-F). We did not detect any difference in sleep between *w^1118^* and *dSERT^4^* and only a slight difference between *w^1118^* and the parent line, with the parent showing a slight decrease in sleep compared to *w^1118^*. Although it is impossible to completely rule out subtle effects caused by genetic background, these results demonstrate disruption of *dSERT* is primarily responsible for the increase in overall sleep and sleep drive we observe in the *dSERT* mutants.”

*3. Serotonin is involved in development of Drosophila. The author should exclude the development effects on sleep phenotype.*

We appreciate the Reviewer’s concern and previous publications have indeed suggested that serotonin may influence fly development, similar to its well described effects in mammals^4–6^. Although the mechanisms remain unclear, a small deletion that includes a 5-HT receptor is embryonic lethal^7^. An increase in serotonergic signaling during development has also been reported to change the morphology of serotonergic processes in larvae^8,9^. Since loss of dSERT may similarly increase serotonergic signaling, we will consider pursuing additional developmental studies in future experiments, and quantitate potential changes in bouton number and/or behavior. For the current manuscript, we suggest that our newly added experiments using the SSRI citalopram address the Reviewer’s concern. Namely, the increase in sleep caused by administration of citalopram to adult flies strongly suggests that a reduction in dSERT activity during adulthood is sufficient to cause a sleep phenotype. By extension, some elements of the *dSERT* mutant phenotype are likely to be caused by loss of dSERT activity during adulthood and after the fly is fully developed. We nonetheless agree with the Reviewer that developmental effects could potentially contribute to the adult sleep phenotype of *dSERT* as well as previously described serotonin receptor mutants. Our future plans include the use of GAL80 or a comparable inducible system to rigorously test this hypothesis. However, these experiments will require a number of months to complete, and we will be unable to include them in the current manuscript without significantly delaying our resubmission beyond the suggested time frame.

*4. For the scatter plot, such as Fig. 2D, Fig. S2A, Fig. 5B, the replicates are not match the number in the brackets, the authors should explain it.*

We have carefully reviewed all of the data, edited the figures and updated the figure legends. We believe that the numbers of replicates we report are now accurate.

*5. For Sup Figure 3C, F, it is more helpful to show whether the mRNA level changed under individual condition other than expression pattern of the TRH-GAL4 and TpH-GAL4. And the expression pattern of TRH-gal4 was so different from previous study (Yongjun Qian, et al., 2017)*

We believe that the Reviewer may be asking to see the mRNA level of *UAS-dSERT*, and that they are suggesting that differences in the level of expression rather than the pattern of expression may be most relevant for understanding the difference between the two serotonergic drivers. Indeed, we do not really know why the two drivers had different behavioral effects and the images we show are not meant to provide a rigorous description of their activity. For the current study, we only wish to show that two established serotonergic drivers have different behavioral effects. A much more extensive study using drivers with different patterns of expression and different levels of expression will be needed to understand which serotonergic neurons regulate sleep, but we reserve this extensive study for a future manuscript.

We do not know for sure why the pattern for the *TRH-GAL4* we used appears different that used in Yongjun Qian, et al., 2017. However, the driver we used was made in the Kravitz lab, and based on our reading of Yongjun Qian, et al., 2017^10^, the driver used in that study may have been remade in the Rao lab. To resolve this issue, it will be necessary to directly compare the two lines. However, these studies would not necessarily change the conclusions of the current manuscript.

***Reviewer 2:***

*1. P(wake)/P(doze) analysis is based on sleep measurement, it helps to understand the arousal threshold and sleep pressure. Thus, the logic for Figure 1 and 2 should be rearranged. In addition, dSERT mutants exhibited higher P(doze) and lower P(wake) than control flies for both day and night, but cannot interpret the differences of sleep structure during the day and at night. No further evidence to better support and illustrate what results in the opposite phenotypes of day and night sleep architecture.*

In response to this suggestion, we have removed the P(wake)/P(doze) analysis from the first figure and have included it instead later in the manuscript as Supplemental Figure 1G-H.

We suggest that some of the new experiments we have included in our revised submission may be relevant to the pathways for day versus night sleep architecture and potentially address the Reviewer’s additional concern. For example, the increased bout number seen during the day is only present in mated female *dSERT^16^* mutants. By contrast, *dSERT^16^* males show a decrease in daytime bout numbers and *dSERT^16^* virgin females do not exhibit an increase in bout number as shown in Supplemental Figure 3C. Interestingly, the post-mating response induced by sex peptide has previously been shown to alter daytime sleep^1^. Based on the differences we observe between mated and unmated females, it is possible that the pathways mediated by sex peptide and serotonin interact. However, further experiments will be needed to test this hypothesis.

The experiments we have added to the revised manuscript testing waking threshold may also be relevant to the Reviewer’s concern. As described in our response to Reviewer 1, our data show an increase in the waking threshold of *dSERT* mutants compared to controls during the night. (Supplemental Figure 4). It is possible that a higher waking threshold and “deeper” sleep could contribute to the consolidated sleep architecture exhibited by *dSERT* mutants at night.

In sum we appreciate the Reviewer’s concern, and some of our data provide hypotheses about the underlying mechanism. It the absence of experiments to test these hypotheses it may be premature to extensively discuss potential mechanisms, but we have added the following text to the Discussion:

“…mated *dSERT* females showed an increase in bout frequency compared to controls but only during the daytime and this effect was not observed in males or virgin females. Sex-peptide also induces changes in daytime sleep in mated females (61) and it is possible that sex-peptide and the post-mating response could modify the effects of *dSERT*.”

*2. Authors attempted to link sexual interaction with sleep by comparing a pair of male and female to a single fly in the DAM system. Since there are other differences such as social effectors or mechanic influences between a single fly and a pair of flies, the conclusion is relatively weak. Would be more convincing to compare a pair of female and male to a pair of females/males.*

We agree and include additional data in our revised manuscript that addresses this concern. As also discussed in our response Reviewer 1, we have tested co-housing with two females as a control (see Figure 5A). The female pairs do not show decreased sleep. These data confirm that the decrease in sleep seen with male-female pairs is not an artifact of housing two flies together, but rather, indicative of courtship and copulation activity.

*3. Food intake was evaluated by starvation of control flies and mutants with the same length of time. As a basic knowledge, the resistance to starvation of each genotype is very different. With the same length of starvation, it possibly results in different starvation status for food intake. Furthermore, it is known that starvation suppresses sleep, but whether less sleep reduction of dSERT mutants is attributed to sleep defects or due to their more resistance to starvation is not conclusive in the present study.*

We appreciate the Reviewer’s insight and agree that sleep, food intake and the effects of starvation are interdependent. We therefore performed additional experiments to compare the resistance to starvation in *dSERT* mutants versus controls (Figure 6E-F). However, we added an additional condition to differentiate between the effects of dehydration/desiccation versus starvation. Our data show that compared to controls, *dSERT* mutants show a decrease in starvation resistance. By contrast, *dSERT* mutants show an increased resistance to desiccation compared to controls. We have incorporated these new findings into the revised manuscript as Figure 6E-F. Since *dSERT* mutants exhibit a decrease in starvation resistance, the decrease in feeding they exhibit is unlikely to be caused by an increased resistance to starvation.

In addition to Figure 6E-F we have added the following text to the revised manuscript:

“We assayed starvation resistance by housing flies on an agar substrate and found that *dSERT* mutants exhibit a significantly decreased probability of survival compared to controls (Figure 6E), with the *dSERT* mutants reaching a 50% mortality rate approximately 15% earlier than the controls.”

and

“In contrast to the effects of starvation, we found that dSERT mutants exhibit a slight increase in resistance to desiccation and survived ~9 % longer than controls (Figure 6F).”

*4. dSERT rescue in two sets of GAL4+ neurons resulted daytime or nighttime specific rescue is interesting. Whether dSERT mutation or application of SSRI can rescue insomnia phenotype, and further rescue in a time specific manner is not conclusive here.*

We appreciate the Reviewer’s comment, but do not fully understand what is meant by “insomnia phenotype” since the phenotype demonstrated in this manuscript is a significant increase in sleep amount. Assuming for the moment that by insomnia they might mean any change in sleep whether increased or decreased, we agree that the timing of the rescue is important. One issue is developmental timing, and whether rescue in adulthood is sufficient to restore wild type sleep. We have not yet used inducible molecular genetic tools to perform developmental versus adult rescue experiments and, as described in our response to Reviewer 1, the time required for these experiments prohibits their inclusion in our revision. However, we have performed additional experiments feeding SSRIs to adult flies and suggest that the results partially address the developmental versus adult effects of dSERT inhibition. While we cannot rule out a developmental component, the increase in sleep caused by an SSRI in the adult indicates that developmental inhibition of dSERT is not necessary for the adult sleep phenotype. To rule out potential off-target effects, we also fed citalopram to the *dSERT* mutant. The absence of an additional effect on sleep and the occlusion of an SSRI effect by the mutant strongly suggest that they are both acting via dSERT.

It is also possible that by “rescue in a time specific manner” the Reviewer is suggesting that we rescue during the day and determine if this also affects the nighttime phenotype (or vice versa). We have found that transporter proteins exhibit significant perdurance (Fei et al and data not shown). Since more than 24 hours would be required to overcome this effect, rapid changes in dSERT expression are unlikely to be feasible.

As suggested by the Reviewer, SSRIs represent an important alternative to molecular-genetic manipulations, and in future experiments it may be possible to apply SSRIs for very short periods of time. However, interpretation of the data would require a more thorough understanding of the pharmacokinetics of citalopram in flies and developing a paradigm in which drug levels were only high enough to block SERT for a circumscribed period of time. Since we do not have this information, we feel that testing the effects of feeding an SSRI only during the day or only at night would not be interpretable at present.

Minor issues:
*1. In the present study, main findings focused on dSERT on sleep accompanied with observations on feeding and courtship behaviors. The title did not properly reflect the main findings. According to this title, one would expect their main findings on the mechanisms of interplay between feeding and courtship behaviors and sleep.*

To better reflect the main findings of the manuscript we have provisionally changed the title to “Mutation of the *Drosophila melanogaster* serotonin transporter *dSERT* impacts sleep, courtship, and feeding behaviors”. However, we would appreciate input on whether we should further modify the title.

*2. Authors used both single-beam and multibeam monitors to assay whether mutation of dSERT results in changes of activity. There is no description of which beam or how they analyzed the data using the multibeam monitors.*

To address this concern, we edited the Materials and Methods section to more clearly describe the analysis. We now indicate that sleep was quantified and analyzed the same way for single beam and multibeam monitors and describe the structure of both types. (Both use tube lengths of 65 mm. The multibeam has 4 independent beams with adjacent beams spaced 9.1 mm apart and the single beam has just one beam in the center of the tube). In brief, in both single beam and multibeam monitors, a single activity count is recorded when a fly breaks the infrared beam. Inactivity requires an absence of any beam breaks and absence of breaks for 5 minutes or more is defined as a sleep bout. Therefore, in both single beam and multibeam monitors, a single beam break negates a sleep bout. The higher number of beams in multibeam monitors increases the chances for activity to be recorded, and results in a more sensitive readout of the fly’s activity within the tube. It is therefore a more conservative measure of sleep.

We have added the following text to the Results:

“To verify that the phenotype was not due to a bias in the time spent on one end of the testing tube (e.g., near the food source) or to limited movements that do not carry the flies past the tube’s midline, we repeated the assays with monitors that use 4 separate beams to detect movement (53). Since multiple sites within the tube are simultaneously assessed for activity, multibeam monitors provide a more sensitive assay, and a more conservative assessment of sleep.”

We have added the following text to the Methods:

“DAM2s were used for single beam monitors and DAM5Ms were used for multibeam monitors (4 independent beams per tube with 9.1 mm adjacent beam spacing).”

*3. The locomotion differences would be better to be evaluated by the activity while awake/the activity while actively moving or using the video tracking analysis.*

We agree with this suggestion and have updated our locomotion data in Figure 4 to show activity while awake (Figure 4C,G). These data indicate that the *dSERT* mutant flies do not have any detectable hyperactivity or locomotion defects and exhibit activity comparable to that of the controls while awake.

We have added the following text to the Results:

“The average activity of *dSERT^16^* flies while awake (activity counts/time awake) did not differ from control flies in LD (Figure 4C) nor DD (Figure 4G).”

*4. The font and standard need to be consistent. A space is needed between the number and the scale/unit. See: Line97, 350, 352, 390, 393, 402, 431, 432, 441-446. Font issue: Line228-229 and Line373-385, used Arial, not consistent through the entire paper; Line422, description of age of flies are not uniformed. Others: i.e. “H2O, 25 C”, Line491, “MH107390, (DEK),”*

We believe that we have corrected these unfortunate inconsistencies within the text.

*5. Many typos: Line 117, “sleep stake”? Line181, “pairing male and females”; Line 245, what does “for” here mean?
6. Citation format is not consistent in the text. i.e. Line 329 “(62)(63)(64)”.*

We thank the Reviewer for helping us to correct these errors, and we have revised the text accordingly.

***Reviewer 3:***

*The main experimental limitation of the entire paper is that the authors missed the opportunity to use the appropriate genetic controls. The process of P-element excision always generates precise or almost-precise excisions that are ideal genetic controls for this type of experiments. The authors report having obtained two of those but did not employ them in any of the comparative studies and* opted *for the outcrossing background w1118 instead. Given that the rescue experiments did not really give a very convincing rescue, using the appropriate controls is a must, at least for some of the key experiments.*

We agree with Reviewer 3 that genetic background is a crucial consideration for all behavioral experiments, and we have also discussed this in our response to Reviewer 1. We have addressed this important concern by comparing sleep behavior in the *w^1118^* control to that of a precise excision line (*dSERT^4^*) obtained in parallel with imprecise excisions *dSERT^10^* and *dSERT^16^* (see Supplemental Figure 1E-F of our revised manuscript). As such, *dSERT^4^*, *dSERT^10^ and dSERT^16^* are all in the same genetic background. We did not detect any difference between sleep behavior of *w^1118^* and *dSERT^4^*. By contrast, *dSERT^16^* sleeps significantly more than both *dSERT^4^ and w^1118^*. We cannot rule out subtle effects of genetic background or architecture on sleep; indeed the parent P line sleeps slightly less than *w^1118^* . However, our data indicate that the genetic background of the excision lines is unlikely to be responsible for the sleep phenotype caused by loss of *dSERT* activity. This conclusion is further reinforced by the effects of the SSRI citalopram on sleep.

The Reviewer also suggests that *“rescue experiments did not really give a very convincing rescue”.* We interpret this to mean that the results were not convincing since each one of the two serotonergic drivers that we tested only rescued daytime sleep or nighttime sleep but not both. In our revised manuscript, we now show additional data indicating that combining the drivers in a single line allows recue of both daytime sleep and nighttime sleep. We are hopeful that this addresses the Reviewer’s concern.

*The other (surely addressable) weakness is that some of the experiments are not performed in the optimal way. For instance, Figures 4A,B and 6I use DAM monitors to assess sleep in co-housed flies and that is really just a very approximate way to detect sleep. For this kind of experiments in which you have multiple flies in the same tube you will have to necessarily use videotracking. However, the results in 4C-G are convincing enough so I would simply remove A and B and describe the finding in more anecdotal terms. 4A and B and 6I - if I understood correctly how the experiment was performed - are just not sound enough to be used as "data".*

As suggested, we have removed 6I from figure 6 (now Figure 7) in the revised manuscript. Regarding Figure 4A, B (now Figure 5A, B), we would like to address the Reviewer’s concern further by providing additional data using video tracking. Unfortunately, we do not have the capability to reliably perform these experiments. We are hopeful that the data we provide using DAMs are acceptable for the revised manuscript.

To account for the potential artifacts caused by housing two flies in the same tube, we have followed the suggestions of the other Reviewers and included new experimental data from female-female overnight pairings. These results indicate that the increased activity that we reported in Figures 4A-B is specific to male-female pairings.

Other suggestions/corrections:

*Line 48: Please add an up-to-date reference for “essential for life”.*

We have added the following reference to the revised manuscript:

Vaccaro A, Dor YK, Nambara K, Pollina EA, Lin C, Greenberg ME, et al. Sleep Loss Can Cause Death through Accumulation of Reactive Oxygen Species in the Gut. Cell. 2020 Jun 11;181(6):1307-1328.e15..

*Line 56: Throughout the paper, please specify Drosophila melanogaster. Drosophila is the name of a genus.*

We have updated the manuscript to specify *Drosophila melanogaste*r within the text.

*L462: the food recipe is actually missing*

We regret this omission and have updated the text to include the food recipe.

*Figure 1C: please do not cut bands from the western blot and show the entire thing, including the molecular weight. This is important for reproducibility.*

We have updated the manuscript to show the full western blot in Figure 1.

*Figure 4A,B and Figure 6I: One cannot measure sleep in co-housed flies using DAMs. The more flies in the tube, the more IR-breaking beams event the system will record. Remove or redo the experiment using videotracking with multiple fly tracking.*

We agree with the Reviewer that more flies in the tube might be expected to cause more IR-beam breaking events than single flies, and that this variable was not well-controlled in our original manuscript. In the revised manuscript, we have added additional controls with two co-housed females to control for the effect of increasing the number of flies per tube. The data is shown in Fig 5 of the revised manuscript (rather than Fig 4 as in the original manuscript). The additional controls with two females are indicated in Fig 5 with dark red bars and “♀+♀” on the x axis.

We do not detect a difference in beam breaks between the tubes that contain a single female compared to those that contain two females. We agree that this result is somewhat counterintuitive. Nonetheless, it serves as a control for the tubes that contain one female and one male and show that the decrease in sleep seen in these pairings is caused by sexual activity rather than simply increasing the number of flies. As suggested by the Reviewer, video tracking the flies would be another way to control for the potential confound caused by co-housing. Unfortunately, we not currently equipped to use this method.

*Figure 4C: rather than cap the assay at one hour, it is more informative to measure how long it takes for copulation to occur (that is, latency as you correctly did for D and E).*

Our decision to cap the assay at one hour is based on experimental previous publications on courtship and copulation behavior^11,12^. While we appreciate the Reviewer’s suggestion, and additional data would be interesting, we do not think that it would change our conclusions.

*Figure 5B,C: feeding assays with dyes are good because they are easily and quickly quantifiable with a spectrophotometry of squashed flies (See: J. exp. Biol. 197, 215–235 (1994)). Here you seem to have adopted a fully subjective system, losing most of the strength of the assay*.

We agree with the Reviewer and think this is a wonderful idea. We therefore repeated our starvation and feeding assays and used spectrophotometry to quantify the absorbance of blue food dye in the squashed flies. This new data has been added to the manuscript as Figure 6D.

**References:**

1.	Isaac, R. E., Li, C., Leedale, A. E. & Shirras, A. D. Drosophila male sex peptide inhibits siesta sleep and promotes locomotor activity in the post-mated female. *Proc. R. Soc. B Biol. Sci.* **277**, 65–70 (2010).

2.	Pooryasin, A. & Fiala, A. Identified Serotonin-Releasing Neurons Induce Behavioral Quiescence and Suppress Mating in Drosophila. *J. Neurosci.* **35**, 12792–12812 (2015).

3.	Liu, C. *et al.* A Serotonin-Modulated Circuit Controls Sleep Architecture to Regulate Cognitive Function Independent of Total Sleep in Drosophila. *Curr. Biol. CB* **29**, 3635-3646.e5 (2019).

4.	Ansorge, M. S., Zhou, M., Lira, A., Hen, R. & Gingrich, J. A. Early-life blockade of the 5-HT transporter alters emotional behavior in adult mice. *Science* **306**, 879–881 (2004).

5.	Shah, R., Courtiol, E., Castellanos, F. X. & Teixeira, C. M. Abnormal Serotonin Levels During Perinatal Development Lead to Behavioral Deficits in Adulthood. *Front. Behav. Neurosci.* **12**, 114 (2018).

6.	Soiza-Reilly, M. *et al.* SSRIs target prefrontal to raphe circuits during development modulating synaptic connectivity and emotional behavior. *Mol. Psychiatry* **24**, 726–745 (2019).

7.	Colas, J.-F., Launay, J.-M. & Maroteaux, L. Maternal and zygotic control of serotonin biosynthesis are both necessary for Drosophila germband extension. *Mech. Dev.* **87**, 67–76 (1999).

8.	Budnik, V., Wu, C. F. & White, K. Altered branching of serotonin-containing neurons in Drosophila mutants unable to synthesize serotonin and dopamine. *J. Neurosci. Off. J. Soc. Neurosci.* **9**, 2866–2877 (1989).

9.	Daubert, E. A., Heffron, D. S., Mandell, J. W. & Condron, B. G. Serotonergic dystrophy induced by excess serotonin. *Mol. Cell. Neurosci.* **44**, 297–306 (2010).

10.	Qian, Y. *et al.* Sleep homeostasis regulated by 5HT2b receptor in a small subset of neurons in the dorsal fan-shaped body of drosophila. *eLife* **6**, e26519 (2017).

11.	Rezával, C., Nojima, T., Neville, M. C., Lin, A. C. & Goodwin, S. F. Sexually Dimorphic Octopaminergic Neurons Modulate Female Postmating Behaviors in Drosophila. *Curr. Biol.* **24**, 725–730 (2014).

12.	Lee, H., Choi, H. W., Zhang, C., Park, Z.-Y. & Kim, Y.-J. A Pair of Oviduct-Born Pickpocket Neurons Important for Egg-Laying in Drosophila melanogaster. *Mol. Cells* **39**, 573–579 (2016).
